# Supplementary material for: Parkinson’s Disease and SARS-CoV-2 Infection: Particularities of Molecular and Cellular Mechanisms Regarding Pathogenesis and Treatment
Source: Biomedicines. 2022 Apr 26;10(5):1000. doi: 10.3390/biomedicines10051000 (PMC9138688; doi:10.3390/biomedicines10051000)
Supplement: Supplementary file 1 [file biomedicines-10-01000-s001.zip › biomedicines-1660777-supplementary.pdf]

**Title:**	**Parkinson's disease and SARS-CoV2 infection: particularities o**		
**Start date:**	**1.1.21**		
**End date:**	**31.12.21**		
**No of keywords**	**26**		
	**Part 1**	**Part 2**	**Part 3**
**Keywords 1**	SARS-CoV-2	nanotechnology	Parkinson
**Keywords 2**	SARS-CoV-2	nanodrugs	Parkinson
**Keywords 3**	SARS-CoV-2	nanomaterials	Parkinson
**Keywords 4**	SARS-CoV-2	Chaperone	Parkinson
**Keywords 5**	SARS-CoV-2	Neurodegeneration	Parkinson
**Keywords 6**	SARS-CoV-2	α-synuclein	Parkinson
**Keywords 7**	SARS-CoV-2	pathophysiology	Parkinson
**Keywords 8**	SARS-CoV-2	Genetic Subtypes	Parkinson
**Keywords 9**	SARS-CoV-2	Neurofilament proteins	Parkinson
**Keywords 10**	SARS-CoV-2	microglia	Parkinson
**Keywords 11**	SARS-CoV-2	misfolded proteins	Parkinson
**Keywords 12**	SARS-CoV-2	pro-inflammatory mediators	Parkinson
**Keywords 13**	Long Covid	Neuroinflammation	Parkinson
**Keywords 14**	Long Covid	Neurodegeneration	Parkinson
**Keywords 15**	COVID-19	nanotechnology	Parkinson
**Keywords 16**	COVID-19	nanodrugs	Parkinson
**Keywords 17**	COVID-19	nanomaterials	Parkinson
**Keywords 18**	COVID-19	Chaperone	Parkinson
**Keywords 19**	COVID-19	Neurodegeneration	Parkinson
**Keywords 20**	COVID-19	α-synuclein	Parkinson
**Keywords 21**	COVID-19	pathophysiology	Parkinson
**Keywords 22**	COVID-19	Genetic Subtypes	Parkinson
**Keywords 23**	COVID-19	Neurofilament proteins	Parkinson
**Keywords 24**	COVID-19	microglia	Parkinson
**Keywords 25**	COVID-19	misfolded proteins	Parkinson
**Keywords 26**	COVID-19	pro-inflammatory mediators	Parkinson

Source	Title	Authors	Extra	Type	Acce	PDF	Keywords				Nr. de citari pe	Nr de referințe
pubmed	COVID-19: dealing with a potential risk factor for chronic neurological disorders.	Schirinzi T, Landi D, Liguori C.	J Neurol. 2021 Apr		Free	https://pubmed.ncbi.nlm.nih.gov//32852580/	"SARS-CoV-2" + "Neurodegeneration" + "Parkinson"				24	84
pubmed	Neuroprotective effects of flavone luteolin in neuroinflammation and neurotrauma.	Kempuraj D, Thangavel R, Kempuraj DD, Ahmed ME, Sel	Biofactors. 2021 M		Free	https://pubmed.ncbi.nlm.nih.gov//33098588/	"SARS-CoV-2" + "Neurodegeneration" + "Parkinson"				22	79
pubmed	Pathophysiological Clues to How the Emergent SARS-CoV-2 Can Potentially Increase t	Dolatshahi M, Sabahi M, Aarabi MH.	Mol Neurobiol. 20		Free	https://pubmed.ncbi.nlm.nih.gov//33417221/	"SARS-CoV-2" + "Neurodegeneration" + "Parkinson"				21	157
pubmed	Microglial Implications in SARS-CoV-2 Infection and COVID-19: Lessons From Viral RN	Awogbindin IO, Ben-Azu B, Olusola BA, Akinluyi ET, Ade	Front Cell Neurosc		Free	https://pubmed.ncbi.nlm.nih.gov//34211370/	"SARS-CoV-2" + "Neurodegeneration" + "Parkinson"				6	286
pubmed	The Intersection of Parkinson's Disease, Viral Infections, and COVID-19.	Rosen B, Kurtishi A, Vazquez-Jimenez GR, Møller SG.	Mol Neurobiol. 20		Free	https://pubmed.ncbi.nlm.nih.gov//34033061/	"SARS-CoV-2" + "Neurodegeneration" + "Parkinson"				4	103
pubmed	Alzheimer's and Parkinson's Diseases Predict Different COVID-19 Outcomes: A UK Bio	Yu Y, Travaglio M, Popovic R, Leal NS, Martins LM.	Geriatrics (Basel).		Free	https://pubmed.ncbi.nlm.nih.gov//33530357/	"SARS-CoV-2" + "Neurodegeneration" + "Parkinson"				25	59
pubmed	Clinical Profiles and Mortality of COVID-19 Inpatients with Parkinson's Disease in Ger	Scherbaum R, Kwon EH, Richter D, Bartig D, Gold R, Krog	Mov Disord. 2021		Free	https://pubmed.ncbi.nlm.nih.gov//33942924/	"SARS-CoV-2" + "Neurodegeneration" + "Parkinson"				11	40
pubmed	Parkinson's Disease and Post-COVID-19 Syndrome: The Parkinson's Long-COVID Spec	Leta V, Rodríguez-Violante M, Abundes A, Rukavina K, T	Mov Disord. 2021		Free	https://pubmed.ncbi.nlm.nih.gov//33890344/	"SARS-CoV-2" + "Neurodegeneration" + "Parkinson"				17	7
pubmed	Vitamin D supplementation and outcomes in coronavirus disease 2019 (COVID-19) pa	Cereda E, Bogliolo L, Lobascio F, Barichella M, Zecchinell	Nutrition. 2021 Fe		Free	https://pubmed.ncbi.nlm.nih.gov//33288411/	"SARS-CoV-2" + "pathophysiology" + "Parkinson"				38	14
pubmed	The intracellular renin-angiotensin system: Friend or foe. Some light from the dopam	Labandeira-Garcia JL, Valenzuela R, Costa-Besada MA, V	Prog Neurobiol. 20		Free	https://pubmed.ncbi.nlm.nih.gov//33039415/	"SARS-CoV-2" + "pathophysiology" + "Parkinson"				4	179
pubmed	Role of astroglial toll-like receptors (TLRs) in central nervous system infections, injury	Li L, Acioglu C, Heary RF, Elkabes S.	Brain Behav Immu		Free	https://pubmed.ncbi.nlm.nih.gov//33039660/	"SARS-CoV-2" + "microglia" + "Parkinson" + "astrocyt				31	276
pubmed	Telehealth in Neurodegenerative Diseases: Opportunities and Challenges for Patients	De Marchi F, Contaldi E, Magistrelli L, Cantello R, Comi C	Brain Sci. 2021 Feb		Free	https://pubmed.ncbi.nlm.nih.gov//33668641/	"COVID-19" + "Neurodegeneration" + "Parkinson"				7	92
pmc	Silver nanoparticles as a potential treatment against SARS-CoV-2: A review	Fernanda Pilaquinga, Jeroni Morey, Marbel Torres, Rach	Wiley Interdiscip R		Free	https://www.ncbi.nlm.nih.gov/pmc/articles/PMC7995207/	"SARS-CoV-2" + "nanotechnology" + "Parkinson"				8	153
pmc	Nanotechnology advances in pathogen- and host-targeted antiviral delivery: multipro	Kai-Chieh Yang, Jung-Chen Lin, Hsiao-Han Tsai, Chung-Ya	Drug Deliv Transl R		Free	https://www.ncbi.nlm.nih.gov/pmc/articles/PMC7982277/	"SARS-CoV-2" + "nanotechnology" + "Parkinson"				6	148
pmc	Applications of nanotechnology in virus detection, tracking, and infection mechanism	Jun Kang, Ayesha Tahir, Hanjie Wang, Jin Chang	Wiley Interdiscip R		Free	https://www.ncbi.nlm.nih.gov/pmc/articles/PMC7995016/	"SARS-CoV-2" + "nanotechnology" + "Parkinson"				4	81
pmc	Electrochemical diagnostics of infectious viral diseases: Trends and challenges	K. Yugender Goud, K. Koteshwara Reddy, Ahmed Khors	Biosens Bioelectro		Free	https://www.ncbi.nlm.nih.gov/pmc/articles/PMC7921732/	"SARS-CoV-2" + "nanotechnology" + "Parkinson"				10	245
pmc	Nanomaterials for the Diagnosis and Treatment of Urinary Tract Infections	Maimoona Qindeel, Mahmood Barani, Abbas Rahdar, Ra	Nanomaterials (Ba		Free	https://www.ncbi.nlm.nih.gov/pmc/articles/PMC7926703/	"SARS-CoV-2" + "nanotechnology" + "Parkinson"				16	164
pmc	Probiotics, Photobiomodulation, and Disease Management: Controversies and Challe	Laura Marinela Ailioaie, Gerhard Litscher	Int J Mol Sci. 2021		Free	https://www.ncbi.nlm.nih.gov/pmc/articles/PMC8124384/	"SARS-CoV-2" + "nanotechnology" + "Parkinson"				9	314
pmc	Nanomaterials for the Diagnosis and Treatment of Inflammatory Arthritis	Seyedeh Maryam Hosseinikhah, Mahmood Barani, Abba	Int J Mol Sci. 2021		Free	https://www.ncbi.nlm.nih.gov/pmc/articles/PMC8002885/	"SARS-CoV-2" + "nanotechnology" + "Parkinson"				8	160
pmc	Toxicology and pharmacology of synthetic organoselenium compounds: an update	Cristina W. Nogueira, Nilda V. Barbosa, João B. T. Rocha	Arch Toxicol. 2021		Free	https://www.ncbi.nlm.nih.gov/pmc/articles/PMC8012418/	"SARS-CoV-2" + "nanotechnology" + "Parkinson"				17	712
pmc	Biochemical, Ameliorative and Cytotoxic Effects of Newly Synthesized Curcumin Micr	Abbas Rahdar, Mohammad Reza Hajinezhad, Saman Sar	Nanomaterials (Ba		Free	https://www.ncbi.nlm.nih.gov/pmc/articles/PMC8004644/	"SARS-CoV-2" + "nanotechnology" + "Parkinson"				6	96
pmc	Integrated network analysis reveals new genes suggesting COVID-19 chronic effects a	Alisa Pavel, Giusy del Giudice, Antonio Federico, Antoni	Brief Bioinform. 20		Free	https://www.ncbi.nlm.nih.gov/pmc/articles/PMC7929418/	"SARS-CoV-2" + "nanomaterials" + "Parkinson"				6	98
pmc	Human iPSC-Based Modeling of Central Nerve System Disorders for Drug Discovery	Lu Qian, Julia TCW	Int J Mol Sci. 2021		Free	https://www.ncbi.nlm.nih.gov/pmc/articles/PMC7865494/	"SARS-CoV-2" + "nanomaterials" + "Parkinson"				7	217
pmc	Electrophysiology Read-Out Tools for Brain-on-Chip Biotechnology	Csaba Forro, Davide Caron, Gian Nicola Angotzi, Vincenz	Micromachines (Ba		Free	https://www.ncbi.nlm.nih.gov/pmc/articles/PMC7912435/	"SARS-CoV-2" + "nanomaterials" + "Parkinson"				6	403
pmc	Evolving AAV-delivered therapeutics towards ultimate cures	Xiangjun He, Brian Anugerah Urip, Zhenjie Zhang, Chun	J Mol Med (Berl) 2		Free	https://www.ncbi.nlm.nih.gov/pmc/articles/PMC7885987/	"SARS-CoV-2" + "nanomaterials" + "Parkinson"				10	228
pmc	Functions of ROS in Macrophages and Antimicrobial Immunity	Marc Herb, Michael Schramm	Antioxidants (Base		Free	https://www.ncbi.nlm.nih.gov/pmc/articles/PMC7923022/	"SARS-CoV-2" + "nanomaterials" + "Parkinson"				30	425
pmc	Therapeutic Potential of Exploiting Autophagy Cascade Against Coronavirus Infection	Subhajit Maity, Abhik Saha	Front Microbiol. 20		Free	https://www.ncbi.nlm.nih.gov/pmc/articles/PMC8160449/	"SARS-CoV-2" + "Chaperone" + "Parkinson"				4	177
pmc	Alpha-synuclein research: defining strategic moves in the battle against Parkinson's d	Luis M. A. Oliveira, Thomas Gasser, Robert Edwards, Ma	NPJ Parkinsons Dis		Free	https://www.ncbi.nlm.nih.gov/pmc/articles/PMC8313662/	"SARS-CoV-2" + "Chaperone" + "Parkinson"				4	326
pmc	Autophagy in major human diseases	Daniel J Klionsky, Giulia Petroni, Ravi K Amaravadi, Eric H	EMBO J. 2021 Oct		Free	https://www.ncbi.nlm.nih.gov/pmc/articles/PMC8488577/	"SARS-CoV-2" + "Chaperone" + "Parkinson"				20	775
pmc	Machinery, regulation and pathophysiological implications of autophagosome matur	Yan G. Zhao, Patrice Codogno, Hong Zhang	Nat Rev Mol Cell B		Free	https://www.ncbi.nlm.nih.gov/pmc/articles/PMC8300085/	"SARS-CoV-2" + "Chaperone" + "Parkinson"				15	225
pmc	Diversity and versatility of p38 kinase signalling in health and disease	Begoña Canovas, Angel R. Nebreda	Nat Rev Mol Cell B		Free	https://www.ncbi.nlm.nih.gov/pmc/articles/PMC7838852/	"SARS-CoV-2" + "Chaperone" + "Parkinson"				45	264
pmc	Human Paraoxonase-2 (PON2): Protein Functions and Modulation	Giuseppe Manco, Elena Porzio, Teresa Maria Carusone	Antioxidants (Base		Free	https://www.ncbi.nlm.nih.gov/pmc/articles/PMC7915308/	"SARS-CoV-2" + "Chaperone" + "Parkinson"				9	204
pmc	Superoxide Dismutase Administration: A Review of Proposed Human Uses	Arianna Carolina Rosa, Daniele Corsi, Niccolò Cavi, Natas	Molecules. 2021 A		Free	https://www.ncbi.nlm.nih.gov/pmc/articles/PMC8037464/	"SARS-CoV-2" + "Chaperone" + "Parkinson"				9	322
pmc	The Endothelium Is Both a Target and a Barrier of HDL's Protective Functions	Jérôme Robert, Elena Osto, Arnold von Eckardstein	Cells. 2021 May; 1		Free	https://www.ncbi.nlm.nih.gov/pmc/articles/PMC8146309/	"SARS-CoV-2" + "Chaperone" + "Parkinson"				9	188
pmc	Atypical p38 Signaling, Activation, and Implications for Disease	Jeremy C. Burton, William Antoniades, Jennifer Okalova	Int J Mol Sci. 2021		Free	https://www.ncbi.nlm.nih.gov/pmc/articles/PMC8073329/	"SARS-CoV-2" + "Chaperone" + "Parkinson"				6	211
pmc	Alzheimer's Disease Pathogenesis: Role of Autophagy and Mitophagy Focusing in Mi	Mehdi Eshraghi, Aida Adlimoghaddam, Amir Mahmood	Int J Mol Sci. 2021		Free	https://www.ncbi.nlm.nih.gov/pmc/articles/PMC8036323/	"SARS-CoV-2" + "Chaperone" + "Parkinson"				13	383
pmc	Pathophysiological Clues to How the Emergent SARS-CoV-2 Can Potentially Increase t	Mahsa Dolatshahi, Mohammadmahdi Sabahi, Mohamm	Mol Neurobiol. 20		Free	https://www.ncbi.nlm.nih.gov/pmc/articles/PMC7791539/	"SARS-CoV-2" + "Neurodegeneration" + "Parkinson"				21	157
pmc	Microglial Implications in SARS-CoV-2 Infection and COVID-19: Lessons From Viral RN	Ifeoluwa O. Awogbindin, Benneth Ben-Azu, Babatunde	Front Cell Neurosc		Free	https://www.ncbi.nlm.nih.gov/pmc/articles/PMC8240959/	"SARS-CoV-2" + "Neurodegeneration" + "Parkinson"				6	235
pmc	Elucidating the Neuropathologic Mechanisms of SARS-CoV-2 Infection	Mar Pacheco-Herrero, Luis O. Soto-Rojas, Charles R. Har	Front Neurol. 2021		Free	https://www.ncbi.nlm.nih.gov/pmc/articles/PMC8072392/	"SARS-CoV-2" + "Neurodegeneration" + "Parkinson"				12	220
pmc	Induced dysregulation of ACE2 by SARS-CoV-2 plays a key role in COVID-19 severity	Maryam Eskandari Mehrabadi, Roohullah Hemmati, Am	Biomed Pharmaco		Free	https://www.ncbi.nlm.nih.gov/pmc/articles/PMC7862910/	"SARS-CoV-2" + "Neurodegeneration" + "Parkinson"				6	245
pmc	Targeting Neurological Manifestations of Coronaviruses by Candidate Phytochemicals	Sajad Fakhri, Sana Piri, Mohammad Bagher Majnooni, M	Front Pharmacol. 2		Free	https://www.ncbi.nlm.nih.gov/pmc/articles/PMC7941749/	"SARS-CoV-2" + "Neurodegeneration" + "Parkinson"				5	265
pmc	Parkinson's Disease and the COVID-19 Pandemic	Conor Fearon, Alfonso Fasano	J Parkinsons Dis. 2		Free	https://www.ncbi.nlm.nih.gov/pmc/articles/PMC8150477/	"SARS-CoV-2" + "Neurodegeneration" + "Parkinson"				13	106
pmc	The Immunopathogenesis of Neuroinvasive Lesions of SARS-CoV-2 Infection in COVII	Shamila D. Alipoor, Esmaeil Mortaz, Mohammad Varahr	Front Neurol. 2021		Free	https://www.ncbi.nlm.nih.gov/pmc/articles/PMC8363128/	"SARS-CoV-2" + "Neurodegeneration" + "Parkinson"				6	124
pmc	Immune mediating molecules and pathogenesis of COVID-19-associated neurologica	HariOm Singh, Amita Singh, Abdul Arif Khan, Vivek Gupt	Microb Pathog. 20		Free	https://www.ncbi.nlm.nih.gov/pmc/articles/PMC8177310/	"SARS-CoV-2" + "Neurodegeneration" + "Parkinson"				4	98
pmc	Viral infections and their relationship to neurological disorders	Jéssica Wouk, Daniele Zendrini Rechenchoski, Bianca Ce	Arch Virol. 2021 Ja		Free	https://www.ncbi.nlm.nih.gov/pmc/articles/PMC7838016/	"SARS-CoV-2" + "Neurodegeneration" + "Parkinson"				14	213
pmc	Impact of COVID-19 Pandemic on Patients With Neurodegenerative Diseases	Chao Hu, Cao Chen, Xiao-Ping Dong	Front Aging Neuro		Free	https://www.ncbi.nlm.nih.gov/pmc/articles/PMC8060506/	"SARS-CoV-2" + "Neurodegeneration" + "Parkinson"				7	73
pmc	Parkinson's Disease and Post–COVID-19 Syndrome: The Parkinson's Long-COVID Spec	Valentina Leta, Mayela Rodríguez-Violante, Arturo Abur	Mov Disord. 2021		Free	https://www.ncbi.nlm.nih.gov/pmc/articles/PMC8250675/	"SARS-CoV-2" + "Neurodegeneration" + "Parkinson"				16	7
pmc	Characterization and Biomarker Analyses of Post-COVID-19 Complications and Neuro	Bing Sun, Norina Tang, Michael J. Peluso, Nikita S. Iyer, I	Cells. 2021 Feb; 10		Free	https://www.ncbi.nlm.nih.gov/pmc/articles/PMC7918597/	"SARS-CoV-2" + "Neurodegeneration" + "Parkinson"				25	57
pmc	COVID-19-Associated Neurological Manifestations: An Emerging Electroencephalogra	Geoffroy Vellieux, Romain Sonneville, Sérafima Vledout	Front Physiol. 2020		Free	https://www.ncbi.nlm.nih.gov/pmc/articles/PMC7933549/	"SARS-CoV-2" + "Neurodegeneration" + "Parkinson"				4	116
pmc	Clinical Profiles and Mortality of COVID-19 Inpatients with Parkinson's Disease in Ger	Raphael Scherbaum, Eun Hae Kwon, Daniel Richter, Dirk	Mov Disord. 2021		Free	https://www.ncbi.nlm.nih.gov/pmc/articles/PMC8207013/	"SARS-CoV-2" + "Neurodegeneration" + "Parkinson"				10	40
pmc	Direct and indirect neurological, cognitive, and behavioral effects of COVID-19 on the	Francesco Iodice, Valeria Cassano, Paolo M. Rossini	Neurol Sci. 2021 Ja		Free	https://www.ncbi.nlm.nih.gov/pmc/articles/PMC7787936/	"SARS-CoV-2" + "Neurodegeneration" + "Parkinson"				14	61
pmc	Engineering organoids	Moritz Hofer, Matthias P. Lutolf	Nat Rev Mater. 20		Free	https://www.ncbi.nlm.nih.gov/pmc/articles/PMC7893133/	"SARS-CoV-2" + "Neurodegeneration" + "Parkinson"				77	277
pmc	The impact of Parkinson's disease on manifestations and outcomes of Covid-19 patie	Amr Ehab El-Qushayri, Sherief Ghozy, Abdullah Reda, Al	Rev Med Virol. 202		Free	https://www.ncbi.nlm.nih.gov/pmc/articles/PMC8420424/	"SARS-CoV-2" + "Neurodegeneration" + "Parkinson"				4	78
pmc	Autoantibodies in neurological disease	Harald Prüss	Nat Rev Immunol.		Free	https://www.ncbi.nlm.nih.gov/pmc/articles/PMC8111372/	"SARS-CoV-2" + "Neurodegeneration" + "Parkinson"				16	202
pmc	Toward Understanding the Molecular Role of SNX27/Retromer in Human Health and	Mintu Chandra, Amy K. Kendall, Lauren P. Jackson	Front Cell Dev Biol		Free	https://www.ncbi.nlm.nih.gov/pmc/articles/PMC8083963/	"SARS-CoV-2" + "Neurodegeneration" + "Parkinson"				4	165
pmc	Dementia is an age-independent risk factor for severity and death in COVID-19 inpati	Ana C. Tahira, Sergio Verjovski-Almeida, Sergio T. Ferrei	Alzheimers Demen		Free	https://www.ncbi.nlm.nih.gov/pmc/articles/PMC8250282/	"SARS-CoV-2" + "Neurodegeneration" + "Parkinson"				7	27
pmc	COVID-19 in adults with dementia: clinical features and risk factors of mortality—a cl	Agathe Vrillon, Elsa Mhanna, Clément Aveneau, Manon	Alzheimers Res Th		Free	https://www.ncbi.nlm.nih.gov/pmc/articles/PMC8035874/	"SARS-CoV-2" + "Neurodegeneration" + "Parkinson"				6	38
pmc	Telehealth in Neurodegenerative Diseases: Opportunities and Challenges for Patients	Fabiola De Marchi, Elena Contaldi, Luca Magistrelli, Robe	Brain Sci. 2021 Feb		Free	https://www.ncbi.nlm.nih.gov/pmc/articles/PMC7917616/	"SARS-CoV-2" + "Neurodegeneration" + "Parkinson"				7	92
pmc	Infection and Risk of Parkinson's Disease	Richard J. Smeyne, Alastair J. Noyce, Matthew Byrne, Ro	J Parkinsons Dis. 2		Free	https://www.ncbi.nlm.nih.gov/pmc/articles/PMC7990414/	"SARS-CoV-2" + "Neurodegeneration" + "Parkinson"				6	110
pmc	Broader Insights into Understanding Tumor Necrosis Factor and Neurodegenerative I	I.A. Clark, B. Vissel	J Alzheimers Dis. 2		Free	https://www.ncbi.nlm.nih.gov/pmc/articles/PMC7990436/	"SARS-CoV-2" + "Neurodegeneration" + "Parkinson"				8	203
pmc	Citrus Polyphenols in Brain Health and Disease: Current Perspectives	Matthew G. Pontifex, Mohammad M. A. H. Malik, Emily	Front Neurosci. 20		Free	https://www.ncbi.nlm.nih.gov/pmc/articles/PMC7933480/	"SARS-CoV-2" + "Neurodegeneration" + "Parkinson"				6	177
pmc	Comprehensive annotation of the Chinese tree shrew genome by large-scale RNA se	Mao-Sen Ye, Jin-Yan Zhang, Dan-Dan Yu, Min Xu, Ling Xu	Zool Res. 2021 No		Free	https://www.ncbi.nlm.nih.gov/pmc/articles/PMC8645884/	"SARS-CoV-2" + "Neurodegeneration" + "Parkinson"				5	111
pmc	Extracellular Vesicles as Biomarkers and Therapeutic Tools: From Pre-Clinical to Clinic	Maria Chiara Ciferri, Rodolfo Quarto, Roberta Tasso	Biology (Basel) 202		Free	https://www.ncbi.nlm.nih.gov/pmc/articles/PMC8145169/	"SARS-CoV-2" + "Neurodegeneration" + "Parkinson"				7	91
pmc	Anthocyanins: Promising Natural Products with Diverse Pharmacological Activities	Jiaqi Liu, Hongbing Zhou, Li Song, Zhanjun Yang, Min Qiu	Molecules. 2021 Ju		Free	https://www.ncbi.nlm.nih.gov/pmc/articles/PMC8270296/	"SARS-CoV-2" + "Neurodegeneration" + "Parkinson"				5	142
pmc	Dementia wellbeing and COVID-19: Review and expert consensus on current researc	Kathy Y. Liu, Robert Howard, Sube Banerjee, Adelina Co	Int J Geriatr Psychi		Free	https://www.ncbi.nlm.nih.gov/pmc/articles/PMC8237017/	"SARS-CoV-2" + "Neurodegeneration" + "Parkinson"				8	145
pmc	Cannabis sativa L. as a Natural Drug Meeting the Criteria of a Multitarget Approach to	Anna Stasiłowicz, Anna Tomala, Irma Podolak, Judyta Ci	Int J Mol Sci. 2021		Free	https://www.ncbi.nlm.nih.gov/pmc/articles/PMC7830475/	"SARS-CoV-2" + "Neurodegeneration" + "Parkinson"				19	153
pmc	Amantadine: reappraisal of the timeless diamond—target updates and novel therape	Wojciech Danysz, Andrzej Dekundy, Astrid Scheschonka	J Neural Transm (V		Free	https://www.ncbi.nlm.nih.gov/pmc/articles/PMC7901515/	"SARS-CoV-2" + "Neurodegeneration" + "Parkinson"				7	343
pmc	Angiotensin receptors and neuropathic pain	Mihály Balogh, Clarissa Aguilar, Nicholas T. Nguyen, And	Pain Rep. 2021; 6(		Free	https://www.ncbi.nlm.nih.gov/pmc/articles/PMC8108581/	"SARS-CoV-2" + "Neurodegeneration" + "Parkinson"				6	145
pmc	Mesenchymal Stem Cells as a Cornerstone in a Galaxy of Intercellular Signals: Basis fo	Silvia Fernández-Francos, Noemi Eiro, Luis A. Costa, Sara	Int J Mol Sci. 2021		Free	https://www.ncbi.nlm.nih.gov/pmc/articles/PMC8036553/	"SARS-CoV-2" + "Neurodegeneration" + "Parkinson"				10	335
pmc	Lithium and Therapeutic Targeting of GSK-3	Melinda E. Snitow, Rahul S. Bhansali, Peter S. Klein	Cells. 2021 Feb; 10		Free	https://www.ncbi.nlm.nih.gov/pmc/articles/PMC7910927/	"SARS-CoV-2" + "Neurodegeneration" + "Parkinson"				7	271
pmc	Therapeutic Targeting of the Complement System: From Rare Diseases to Pandemics	Peter Garred, Andrea J. Tenner, Tom E. Mollnes	Pharmacol Rev. 20		Free	https://www.ncbi.nlm.nih.gov/pmc/articles/PMC7956994/	"SARS-CoV-2" + "Neurodegeneration" + "Parkinson"				18	423
pmc	Potential of Sulforaphane as a Natural Immune System Enhancer: A Review	Andrea Mahn, Antonio Castillo	Molecules. 2021 F		Free	https://www.ncbi.nlm.nih.gov/pmc/articles/PMC7867070/	"SARS-CoV-2" + "Neurodegeneration" + "Parkinson"				9	71
pmc	Architecture and mechanism of metazoan retromer:SNX3 tubular coat assembly	Natalya Leneva, Oleksiy Kovtun, Dustin R. Morado, John A. G	Sci Adv. 2021 Mar; 7		Free	https://www.ncbi.nlm.nih.gov/pmc/articles/PMC7990337/	"SARS-CoV-2" + "Neurodegeneration" + "Parkinson"				14	70
pmc	Virtual Reality as a Technological-Aided Solution to Support Communication in Persons With	Fabrizio Stasolla, Marta Matamala-Gomez, Sara Bernini, Ales	Front Public Health.		Free	https://www.ncbi.nlm.nih.gov/pmc/articles/PMC7921156/	"SARS-CoV-2" + "Neurodegeneration" + "Parkinson"				8	117
pmc	Atopic dermatitis: an expanding therapeutic pipeline for a complex disease	Thomas Bieber	Nat Rev Drug Discov		Free	https://www.ncbi.nlm.nih.gov/pmc/articles/PMC8377708/	"SARS-CoV-2" + "Neurodegeneration" + "Parkinson"				6	262
pmc	Relevance of Peroxisome Proliferator Activated Receptors in Multitarget Paradigm Associat	Ana Lago-Fernandez, Sara Zarzo-Arias, Nadine Jagerovic, Pau	Int J Mol Sci. 2021 F		Free	https://www.ncbi.nlm.nih.gov/pmc/articles/PMC7863932/	"SARS-CoV-2" + "Neurodegeneration" + "Parkinson"				6	229
pmc	Interplay of Good Bacteria and Central Nervous System: Cognitive Aspects and Mechanistic (	Mahmoud Salami	Front Neurosci. 202		Free	https://www.ncbi.nlm.nih.gov/pmc/articles/PMC7904897/	"SARS-CoV-2" + "Neurodegeneration" + "Parkinson"				4	388
pmc	How COVID-19 will boost remote exercise-based treatment in Parkinson's disease: a narrativ	Agnes Langer, Lucia Gassner, Anna Flotz, Sebastian Hasenaue	NPJ Parkinsons Dis.		Free	https://www.ncbi.nlm.nih.gov/pmc/articles/PMC7940641/	"SARS-CoV-2" + "α-synuclein" + "Parkinson"				11	133
pmc	TLR Agonists as Vaccine Adjuvants Targeting Cancer and Infectious Diseases	Marina Luchner, Sören Reinke, Anita Milicic	Pharmaceutics. 202		Free	https://www.ncbi.nlm.nih.gov/pmc/articles/PMC7911620/	"SARS-CoV-2" + "α-synuclein" + "Parkinson"				17	101
pmc	Advances in TRP channel drug discovery: from target validation to clinical studies	Ari-Pekka Koivisto, Maria G. Belvisi, Rachelle Gaudet, Arpad	Nat Rev Drug Discov		Free	https://www.ncbi.nlm.nih.gov/pmc/articles/PMC8442523/	"SARS-CoV-2" + "α-synuclein" + "Parkinson"				4	304
pmc	Disease-Induced Changes in Salivary Gland Function and the Composition of Saliva	G.B. Proctor, A.M. Shaalan	J Dent Res. 2021 Oc		Free	https://www.ncbi.nlm.nih.gov/pmc/articles/PMC8461045/	"SARS-CoV-2" + "α-synuclein" + "Parkinson"				4	62
pmc	Histopathological findings and clinicopathologic correlation in COVID-19: a systematic review	Stefania Caramaschi, Meghan E. Kapp, Sara E. Miller, Rosana	Mod Pathol. 2021 M		Free	https://www.ncbi.nlm.nih.gov/pmc/articles/PMC8141548/	"SARS-CoV-2" + "pathophysiology" + "Parkinson"				17	201
pmc	Epidemiology and organ specific sequelae of post-acute COVID19: A narrative review	Eleni Korompoki, Maria Gavriatopoulou, Rachel S Hicklen, Io	J Infect. 2021 Jul; 83		Free	https://www.ncbi.nlm.nih.gov/pmc/articles/PMC8118709/	"SARS-CoV-2" + "pathophysiology" + "Parkinson"				27	182
pmc	A comprehensive review of imaging findings in COVID-19 - status in early 2021	Ali Afshar-Oromieh, Helmut Prosch, Cornelia Schaefer-Proko	Eur J Nucl Med Mol		Free	https://www.ncbi.nlm.nih.gov/pmc/articles/PMC8087891/	"SARS-CoV-2" + "pathophysiology" + "Parkinson"				9	313
pmc	Severe COVID-19 is marked by dysregulated serum levels of carboxypeptidase A3 and seroto	Rodolfo Soria-Castro, Yatsiri G. Meneses-Preza, Gloria M. Ro	J Leukoc Biol. 2021		Free	https://www.ncbi.nlm.nih.gov/pmc/articles/PMC8242632/	"SARS-CoV-2" + "pathophysiology" + "Parkinson"				7	38
pmc	Autonomic dysfunction in post-COVID patients with and witfhout neurological symptoms: a p	Alex Buoite Stella, Giovanni Furlanis, Nicolò Arjuna Frezza, R	J Neurol. 2021 Aug		Free	https://www.ncbi.nlm.nih.gov/pmc/articles/PMC8359764/	"SARS-CoV-2" + "pathophysiology" + "Parkinson"				5	77
pmc	Viral vector platforms within the gene therapy landscape	Jote T. Bulcha, Yi Wang, Hong Ma, Phillip W. L. Tai, Guangping	Signal Transduct Tar		Free	https://www.ncbi.nlm.nih.gov/pmc/articles/PMC7868676/	"SARS-CoV-2" + "pathophysiology" + "Parkinson"				64	326
pmc	COVID-19 cognitive deficits after respiratory assistance in the subacute phase: A COVID-reha	Federica Alemanno, Elise Houdayer, Anna Parma, Alfio Spina	PLoS One. 2021; 16(		Free	https://www.ncbi.nlm.nih.gov/pmc/articles/PMC7870071/	"SARS-CoV-2" + "pathophysiology" + "Parkinson"				44	45
pmc	Patient-Led Research Collaborative: embedding patients in the Long COVID narrative	Lisa McCorkell, Gina S. Assaf, Hannah E. Davis, Hannah Wei, A	Pain Rep. 2021; 6(1)		Free	https://www.ncbi.nlm.nih.gov/pmc/articles/PMC8112577/	"SARS-CoV-2" + "pathophysiology" + "Parkinson"				8	43
pmc	Neurological features and outcome in COVID-19: dementia can predict severe disease	Mehran Ghaffari, Hossein Ansari, Nahid Beladimoghadam, Se	J Neurovirol. 2021 J		Free	https://www.ncbi.nlm.nih.gov/pmc/articles/PMC7792552/	"SARS-CoV-2" + "pathophysiology" + "Parkinson"				13	35
pmc	Cytokine and interleukin profile in patients with headache and COVID-19: A pilot, CASE-contr	Javier Trigo, David García-Azorín, Álvaro Sierra-Mencía, Álva	J Headache Pain. 20		Free	https://www.ncbi.nlm.nih.gov/pmc/articles/PMC8177251/	"SARS-CoV-2" + "pathophysiology" + "Parkinson"				9	41
pmc	Telemedicine Use and Health-Related Concerns of Patients With Chronic Conditions During C	Lindsey Nicole Horrell, Sara Hayes, Leslie Beth Herbert, Katie	J Med Internet Res.		Free	https://www.ncbi.nlm.nih.gov/pmc/articles/PMC7894395/	"SARS-CoV-2" + "pathophysiology" + "Parkinson"				6	26
pmc	Bladder Cancer Immunotherapy by BCG Is Associated with a Significantly Reduced Risk of Alz	Danielle Klinger, Brian L. Hill, Noam Barda, Eran Halperin, Of	Vaccines (Basel) 202		Free	https://www.ncbi.nlm.nih.gov/pmc/articles/PMC8151667/	"SARS-CoV-2" + "microglia" + "Parkinson" + "astrocytes"				4	73
pmc	Lactoferrin: A Glycoprotein Involved in Immunomodulation, Anticancer, and Antimicrobial Pr	Quintín Rascón-Cruz, Edward A. Espinoza-Sánchez, Tania S. Si	Molecules. 2021 Jan		Free	https://www.ncbi.nlm.nih.gov/pmc/articles/PMC7795860/	"SARS-CoV-2" + "pro-inflammatory mediators" + "Parkins				15	144
pmc	N-Acetylcysteine: A Review of Clinical Usefulness (an Old Drug with New Tricks)	Gerry K. Schwalfenberg	J Nutr Metab. 2021;		Free	https://www.ncbi.nlm.nih.gov/pmc/articles/PMC8211525/	"SARS-CoV-2" + "pro-inflammatory mediators" + "Parkins				6	150
pmc	Nanotechnology in Bladder Cancer: Diagnosis and Treatment	Mahmood Barani, Seyedeh Maryam Hosseinikhah, Abbas Ra	Cancers (Basel) 202		Free	https://www.ncbi.nlm.nih.gov/pmc/articles/PMC8125468/	"COVID-19" + "nanotechnology" + "Parkinson"				11	166
pmc	Naringenin Nano-Delivery Systems and Their Therapeutic Applications	Mohammed Bhia, Mahzad Motallebi, Banafshe Abadi, Atefel	Pharmaceutics. 202		Free	https://www.ncbi.nlm.nih.gov/pmc/articles/PMC7926828/	"COVID-19" + "nanotechnology" + "Parkinson"				18	181
pmc	Recent advances in wearable sensors and portable electronics for sleep monitoring	Shinjae Kwon, Hojoong Kim, Woon-Hong Yeo	iScience. 2021 May		Free	https://www.ncbi.nlm.nih.gov/pmc/articles/PMC8113882/	"COVID-19" + "nanotechnology" + "Parkinson"				5	72
pmc	Microneedle-based devices for point-of-care infectious disease diagnostics	Rachael V. Dixon, Eldhose Skaria, Wing Man Lau, Philip Mann	Acta Pharm Sin B. 20		Free	https://www.ncbi.nlm.nih.gov/pmc/articles/PMC8206489/	"COVID-19" + "nanotechnology" + "Parkinson"				5	194
pmc	New Neuroprotective Effect of Lemon IntegroPectin on Neuronal Cellular Model	Domenico Nuzzo, Pasquale Picone, Costanza Giardina, Miriam	Antioxidants (Basel)		Free	https://www.ncbi.nlm.nih.gov/pmc/articles/PMC8145755/	"COVID-19" + "nanotechnology" + "Parkinson"				5	59
pmc	Role of lysosomes in physiological activities, diseases, and therapy	Ziqi Zhang, Pengfei Yue, Tianqi Lu, Yang Wang, Yuquan Wei, X	J Hematol Oncol. 20		Free	https://www.ncbi.nlm.nih.gov/pmc/articles/PMC8120021/	"COVID-19" + "nanomaterials" + "Parkinson"				5	430
pmc	Selective autophagy of intracellular organelles: recent research advances	Wen Li, Pengcheng He, Yuge Huang, Yi-Fang Li, Jiahong Lu, M	Theranostics. 2021;		Free	https://www.ncbi.nlm.nih.gov/pmc/articles/PMC7681076/	"COVID-19" + "Chaperone" + "Parkinson"				22	396
pmc	From purines to purinergic signalling: molecular functions and human diseases	Zhao Huang, Na Xie, Peter Illes, Francesco Di Virgilio, Hennin	Signal Transduct Tar		Free	https://www.ncbi.nlm.nih.gov/pmc/articles/PMC8079716/	"COVID-19" + "Chaperone" + "Parkinson"				20	311
pmc	The revival of dithiocarbamates: from pesticides to innovative medical treatments	Laurine Kaul, Regine Süss, Andrew Zannettino, Katharina Ricl	iScience. 2021 Feb 1		Free	https://www.ncbi.nlm.nih.gov/pmc/articles/PMC7868997/	"COVID-19" + "Chaperone" + "Parkinson"				6	102
pmc	Mitochondrial impairments in aetiopathology of multifactorial diseases: common origin but	Lenka Koklesova, Marek Samec, Alena Liskova, Kevin Zhai, D	EPMA J. 2021 Mar;		Free	https://www.ncbi.nlm.nih.gov/pmc/articles/PMC7931170/	"COVID-19" + "Neurodegeneration" + "Parkinson"				14	162
pmc	Ceramide Metabolism and Parkinson's Disease—Therapeutic Targets	Antía Custodia, Marta Aramburu-Núñez, Clara Correa-Paz, Ad	Biomolecules. 2021		Free	https://www.ncbi.nlm.nih.gov/pmc/articles/PMC8301871/	"COVID-19" + "Neurodegeneration" + "Parkinson"				4	218
pmc	Ergothioneine, recent developments	Irwin K. Cheah, Barry Halliwell	Redox Biol. 2021 Ju		Free	https://www.ncbi.nlm.nih.gov/pmc/articles/PMC8113028/	"COVID-19" + "Neurodegeneration" + "Parkinson"				11	177
pmc	Mitochondriopathies as a Clue to Systemic Disorders—Analytical Tools and Mitigating Meas	Alena Liskova, Marek Samec, Lenka Koklesova, Erik Kudela, I	Int J Mol Sci. 2021 F		Free	https://www.ncbi.nlm.nih.gov/pmc/articles/PMC7922866/	"COVID-19" + "Neurodegeneration" + "Parkinson"				10	174
pmc	The Muscle-Brain Axis and Neurodegenerative Diseases: The Key Role of Mitochondria in Ex	Johannes Burtscher, Grégoire P. Millet, Nicolas Place, Bengt	Int J Mol Sci. 2021 J		Free	https://www.ncbi.nlm.nih.gov/pmc/articles/PMC8235687/	"COVID-19" + "Neurodegeneration" + "Parkinson"				8	257
pmc	Scan Once, Analyse Many: Using Large Open-Access Neuroimaging Datasets to Understand th	Christopher R. Madan	Neuroinformatics. 2		Free	https://www.ncbi.nlm.nih.gov/pmc/articles/PMC8111663/	"COVID-19" + "Neurodegeneration" + "Parkinson"				4	294
pmc	Cognitive and behavioural flexibility: neural mechanisms and clinical considerations	Lucina Q. Uddin	Nat Rev Neurosci. 2		Free	https://www.ncbi.nlm.nih.gov/pmc/articles/PMC7856857/	"COVID-19" + "Neurodegeneration" + "Parkinson"				43	148
pmc	The plasma peptides of Alzheimer's disease	Angelique Florentinus-Mefailoski, Peter Bowden, Philip Sche	Clin Proteomics. 202		Free	https://www.ncbi.nlm.nih.gov/pmc/articles/PMC8240224/	"COVID-19" + "Neurodegeneration" + "Parkinson"				335	165
pmc	Kinase drug discovery 20 years after imatinib: progress and future directions	Philip Cohen, Darren Cross, Pasi A. Jänne	Nat Rev Drug Discov		Free	https://www.ncbi.nlm.nih.gov/pmc/articles/PMC8127496/	"COVID-19" + "Neurodegeneration" + "Parkinson"				50	228
pmc	Mitochondrial Metabolism as Target of the Neuroprotective Role of Erythropoietin in Parkin	Federica Rey, Sara Ottolenghi, Toniella Giallongo, Alice Balsa	Antioxidants (Basel)		Free	https://www.ncbi.nlm.nih.gov/pmc/articles/PMC7830512/	"COVID-19" + "Neurodegeneration" + "Parkinson"				10	83
pmc	Cross-Reactivity and Sequence Homology Between Alpha-Synuclein and Food Products: A Ste	Aristo Vojdani, Aaron Lerner, Elroy Vojdani	Cells. 2021 May; 10		Free	https://www.ncbi.nlm.nih.gov/pmc/articles/PMC8147930/	"COVID-19" + "Neurodegeneration" + "Parkinson"				6	102
pmc	Gut Microbiota Interaction with the Central Nervous System throughout Life	Jorge Ojeda, Ariel Ávila, Pía M. Vidal	J Clin Med. 2021 Ma		Free	https://www.ncbi.nlm.nih.gov/pmc/articles/PMC8004117/	"COVID-19" + "Neurodegeneration" + "Parkinson"				13	296
pmc	Advances and Perspectives in Dental Pulp Stem Cell Based Neuroregeneration Therapies	Jon Luzuriaga, Yurena Polo, Oier Pastor-Alonso, Beatriz Pardo	Int J Mol Sci. 2021 A		Free	https://www.ncbi.nlm.nih.gov/pmc/articles/PMC8036729/	"COVID-19" + "Neurodegeneration" + "Parkinson"				5	183
pmc	The importance of ongoing international surveillance for Creutzfeldt–Jakob disease	Neil Watson, Jean-Philippe Brandel, Alison Green, Peter Hern	Nat Rev Neurol. 202		Free	https://www.ncbi.nlm.nih.gov/pmc/articles/PMC8109225/	"COVID-19" + "Neurodegeneration" + "Parkinson"				8	288
pmc	Aging and age-related diseases: from mechanisms to therapeutic strategies	Zhe Li, Zhenkun Zhang, Yikun Ren, Yingying Wang, Jiarui Fang	Biogerontology. 202		Free	https://www.ncbi.nlm.nih.gov/pmc/articles/PMC7838467/	"COVID-19" + "Neurodegeneration" + "Parkinson"				18	231
pmc	The validation status of blood biomarkers of amyloid and phospho-tau assessed with the 5-p	N. J. Ashton, A. Leuzy, T. K. Karikari, N. Mattsson-Carlgren, A.	Eur J Nucl Med Mol		Free	https://www.ncbi.nlm.nih.gov/pmc/articles/PMC8175325/	"COVID-19" + "Neurodegeneration" + "Parkinson"				22	133
pmc	News about the Role of Fluid and Imaging Biomarkers in Neurodegenerative Diseases	Jacopo Meldolesi	Biomedicines. 2021		Free	https://www.ncbi.nlm.nih.gov/pmc/articles/PMC7999537/	"COVID-19" + "Neurodegeneration" + "Parkinson"				5	62
pmc	Electronic Health Interventions in the Case of Multiple Sclerosis: From Theory to Practice	Maria Scholz, Rocco Haase, Dirk Schriefer, Isabel Voigt, Tjalf	Brain Sci. 2021 Feb;		Free	https://www.ncbi.nlm.nih.gov/pmc/articles/PMC7913051/	"COVID-19" + "Neurodegeneration" + "Parkinson"				16	98
pmc	Treatment Options for Motor and Non-Motor Symptoms of Parkinson's Disease	Frank C. Church	Biomolecules. 2021		Free	https://www.ncbi.nlm.nih.gov/pmc/articles/PMC8074325/	"COVID-19" + "Neurodegeneration" + "Parkinson"				9	158
pmc	Paracellular and Transcellular Leukocytes Diapedesis Are Divergent but Interconnected Evolutio	Michel-Edwar Mickael, Norwin Kubick, Pavel Klimovich, Patrick	Genes (Basel) 2021		Free	https://www.ncbi.nlm.nih.gov/pmc/articles/PMC7916592/	"COVID-19" + "Neurodegeneration" + "Parkinson"				5	89
pmc	Parkinson's Disease Drug Therapies in the Clinical Trial Pipeline: 2021 Update	Kevin McFarthing, Gary Rafaloff, Marco A.S. Baptista, Richard I	J Parkinsons Dis. 202		Free	https://www.ncbi.nlm.nih.gov/pmc/articles/PMC8461678/	"COVID-19" + "α-synuclein" + "Parkinson"				5	22
pmc	Stress and mindfulness in Parkinson's disease – a survey in 5000 patients	Anouk van der Heide, Anne E. M. Speckens, Marjan J. Meinder	NPJ Parkinsons Dis.		Free	https://www.ncbi.nlm.nih.gov/pmc/articles/PMC7813889/	"COVID-19" + "α-synuclein" + "Parkinson"				7	62
pmc	Extracellular Vesicles in Blood: Sources, Effects, and Applications	Ainhoa Alberro, Leire Iparraguirre, Adelaide Fernandes, David (	Int J Mol Sci. 2021 A		Free	https://www.ncbi.nlm.nih.gov/pmc/articles/PMC8347110/	"COVID-19" + "α-synuclein" + "Parkinson"				6	183
pmc	Coordinated Reset Vibrotactile Stimulation Induces Sustained Cumulative Benefits in Parkinson'	Kristina J. Pfeifer, Justus A. Kromer, Alexander J. Cook, Traci Ho	Front Physiol. 2021;		Free	https://www.ncbi.nlm.nih.gov/pmc/articles/PMC8055937/	"COVID-19" + "α-synuclein" + "Parkinson"				7	129
pmc	The Implications of ncRNAs in the Development of Human Diseases	Elena López-Jiménez, Eduardo Andrés-León	Noncoding RNA. 202		Free	https://www.ncbi.nlm.nih.gov/pmc/articles/PMC8006041/	"COVID-19" + "α-synuclein" + "Parkinson"				9	189
pmc	Proceedings of the Eighth Annual Deep Brain Stimulation Think Tank: Advances in Optogenetics	Vinata Vedam-Mai, Karl Deisseroth, James Giordano, Gabriel L	Front Hum Neurosci.		Free	https://www.ncbi.nlm.nih.gov/pmc/articles/PMC8092047/	"COVID-19" + "pathophysiology" + "Parkinson"				10	128
pmc	Rapid Onset Functional Tic-Like Behaviors in Young Females During the COVID-19 Pandemic	Tamara Pringsheim, Christos Ganos, Joseph F. McGuire, Tamm	Mov Disord. 2021 Au		Free	https://www.ncbi.nlm.nih.gov/pmc/articles/PMC8441698/	"COVID-19" + "pathophysiology" + "Parkinson"				7	25
pmc	Rotigotine Transdermal Patch for Motor and Non-motor Parkinson's Disease: A Review of 12 Ye	Vanessa Raeder, Iro Boura, Valentina Leta, Peter Jenner, Heinz	CNS Drugs. 2021; 35		Free	https://www.ncbi.nlm.nih.gov/pmc/articles/PMC7871129/	"COVID-19" + "pathophysiology" + "Parkinson"				5	144
pmc	Stress and circulating cell-free mitochondrial DNA: A systematic review of human studies, physi	Caroline Trumpff, Jeremy Michelson, Claudia J. Lagranha, Vero	Mitochondrion. Auth		Free	https://www.ncbi.nlm.nih.gov/pmc/articles/PMC8418815/	"COVID-19" + "pathophysiology" + "Parkinson"				12	197
pmc	Comorbidities of primary headache disorders: a literature review with meta-analysis	Valeria Caponnetto, Manuela Deodato, Micaela Robotti, Maria	J Headache Pain. 202		Free	https://www.ncbi.nlm.nih.gov/pmc/articles/PMC8278743/	"COVID-19" + "pathophysiology" + "Parkinson"				5	201
pmc	Deep brain stimulation of the Cuneiform nucleus for levodopa-resistant freezing of gait in Parki	Stephano J. Chang, Iahn Cajigas, James D. Guest, Brian R. Noga	Pilot Feasibility Stud.		Free	https://www.ncbi.nlm.nih.gov/pmc/articles/PMC8169408/	"COVID-19" + "pathophysiology" + "Parkinson"				7	54
pmc	Targeting oxidative stress in disease: promise and limitations of antioxidant therapy	Henry Jay Forman, Hongqiao Zhang	Nat Rev Drug Discov		Free	https://www.ncbi.nlm.nih.gov/pmc/articles/PMC8243062/	"COVID-19" + "pathophysiology" + "Parkinson"				58	295
pmc	Reactive Oxygen Species: Not Omnipresent but Important in Many Locations	Marc Herb, Alexander Gluschko, Michael Schramm	Front Cell Dev Biol. 2		Free	https://www.ncbi.nlm.nih.gov/pmc/articles/PMC8452931/	"COVID-19" + "pathophysiology" + "Parkinson"				5	275

ClinicalTrials.gov Search Results 03/15/2022

	NCT Number	Title	Status	Study Results	Conditions	Interventions	Characteristics	Population
1	NCT04466839	Evaluation of the Containment Impact Linked to the Covid-19 Pandemic in a Population of Parkinson Patients	Completed	No Results Available	•Parkinson Disease<br>•COVID	•Other: Questionnaire and interview	Study Type: Observational	Enrollment: 411
2	NCT04422353	Video Dance Class and Unsupervised Physical Activity During Covid-19 Pandemic in People With Parkinson's Disease	Recruiting	No Results Available	•Parkinson Disease<br>•Parkinson Disease 10	•Other: Video Dance classes<br>•Other: Unsupervised physical activities	Study Type: Interventional	Enrollment: 60
3	NCT04565080	Clinical Correlates of COVID-19 Pandemic in Patients With Functional Movement Disorder (FMD) and Parkinson's Disease (PD)	Completed	No Results Available	•Parkinson's Disease<br>•Functional Movement Disorders<br>•COVID-19		Study Type: Observational	Enrollment: 44
4	NCT04798066	HBPCOV01:"Intermediate Size Expanded Access Protocol for the Treatment of Post-COVID-19 Syndrome" HBPD05: "Intermediate Size Patient Population Expanded Access IND for the Treatment of Patients With Parkinson's Disease"	Available	No Results Available	•Post COVID-19 Syndrome<br>•Parkinson Disease	•Biological: HB-adMSCs	Study Type: Expanded Access<br>•Intermediate-size Population	
5	NCT04535297	Consequences of the COVID-19 Lockdown on Health and Well-being of Patients With Parkinson Disease and Post-stroke	Completed	No Results Available	•Parkinson Disease<br>•Stroke	•Other: exposure	Study Type: Observational	Enrollment: 198
6	NCT04942392	Digital Dance for People With Parkinson's Disease During the COVID-19 Pandemic	Completed	No Results Available	•Parkinson Disease	•Device: Digital dance for PD	Study Type: Interventional	Enrollment: 33
7	NCT05074771	At Home REhabilitation and Monitoring of People in poST-covid Condition Through ARc-inTellicare Platform (RESTART/RICOMINCIARE)	Enrolling by invitation	No Results Available	•Post-COVID19<br>•Parkinson Disease	•Device: ARC intellicare	Study Type: Interventional	Enrollment: 20
8	NCT04720118	Parkinson's Disease and Experiences Throughout the COVID-19 Pandemic	Recruiting	No Results Available	•Parkinson Disease<br>•Covid19<br>•Satisfaction, Patient<br>•Quality of Life	•Other: No Intervention	Study Type: Observational	Enrollment: 150
9	NCT04982887	Tele-Rehabilitation in Parkinson's Disease	Enrolling by invitation	No Results Available	•Parkinson Disease	•Other: Exercise	Study Type: Interventional	Enrollment: 12
10	NCT04719468	PD-Ballet: Effectiveness and Implementation in Parkinson's Disease	Recruiting	No Results Available	•Parkinson Disease	•Other: Dance with ballet elements	Study Type: Interventional	Enrollment: 160
11	NCT04963894	Effects of Home Rehabilitation of Balance Based on Functional Exercises in People With Parkinson's Disease	Recruiting	No Results Available	•Parkinson Disease	•Other: Home functional balance physiotherapy<br>•Other: Conventional physiotherapy	Study Type: Interventional	Enrollment: 112
12	NCT03944447	Outcomes Mandate National Integration With Cannabis as Medicine for Prevention and Treatment of COVID-19	Recruiting	No Results Available	•Chronic Pain<br>•Chronic Pain Syndrome<br>•Chronic Pain Due to Injury<br>•Chronic Pain Due to Trauma<br>•Fibromyalgia<br>•Seizures<br>•Hepatitis C<br>•Cancer<br>•Crohn Disease<br>•HIV/AIDS<br>•and 23 more	•Drug: Cannabis, Medical<br>•Device: RYAH-Medtech Inhaler	Study Type: Interventional	Enrollment: 200000

	NCT Number	Title	Status	Study Results	Conditions	Interventions	Characteristics	Population
13	NCT04644367	Effects of a Biomechanical-based Tai Chi Program on Gait and Posture in People With Parkinson's Disease	Active, not recruiting	No Results Available	•Parkinson Disease	•Other: Tai Chi intervention<br>•Other: Regular Physical Activity (control) group	Study Type: Interventional	Enrollment: 40
14	NCT04833088	Exploratory Study About the Implementation of Technology in the Rehabilitation	Enrolling by invitation	No Results Available	•Stroke<br>•Parkinson Disease<br>•Covid19<br>•Neglect, Hemispatial	•Other: Focus group discussions	Study Type: Interventional	Enrollment: 100
15	NCT04568707	Evaluation of Biological Response to SARS-COV2 (COVID-19) in Patients With Pre-existing Neurological Disease or Newly Neurological Symptoms (BIO-COCO-NEUROSCIENCES)	Recruiting	No Results Available	•Covid19<br>•Neurologic Manifestations<br>•Psychiatric Manifestations	•Other: blood sample	Study Type: Interventional	Enrollment: 200
16	NCT04782518	Personalizing Exercise for Parkinson Disease	Recruiting	No Results Available	•Parkinson Disease		Study Type: Observational	Enrollment: 1000
17	NCT03797378	Movement-2-Music: Lakeshore Examination of Activity, Disability, and Exercise Response Study	Recruiting	No Results Available	•Spinal Cord Injuries<br>•Traumatic Brain Injury<br>•Spina Bifida<br>•Cerebral Palsy<br>•Stroke<br>•Parkinson Disease<br>•Multiple Sclerosis	•Other: eM2M	Study Type: Interventional	Enrollment: 108
